# Serum Hepatic Enzyme Activity and Alcohol Drinking Status in Relation to the Prevalence of Metabolic Syndrome in the General Japanese Population

**DOI:** 10.1371/journal.pone.0095981

**Published:** 2014-04-22

**Authors:** Hirokazu Uemura, Sakurako Katsuura-Kamano, Miwa Yamaguchi, Fusakazu Sawachika, Kokichi Arisawa

**Affiliations:** Department of Preventive Medicine, Institute of Health Biosciences, The University of Tokushima Graduate School, Tokushima, Japan; Yonsei University College of Medicine, Republic of Korea

## Abstract

**Background:**

Studies on the combined associations of elevated serum hepatic enzyme activity and alcohol drinking with metabolic syndrome are rare. Our objectives were to evaluate the associations of elevated serum hepatic enzyme activity with the prevalence of metabolic syndrome in the general Japanese population and whether alcohol drinking had a modifying effect on these associations.

**Methods:**

We conducted a cross-sectional study with 1,027 men and 1,152 women throughout Japan during 2002–2010. Biochemical factors including alanine aminotransferase (ALT) and gamma-glutamyl transferase (GGT) were determined in overnight fasting blood, and a survey on lifestyle was conducted by questionnaire. Serum ALT and GGT levels were divided into tertiles in men and women, and their associations with the prevalence of metabolic syndrome were evaluated by logistic regressions.

**Results:**

Elevated serum ALT and GGT, even within the reference range, were independently associated with increased metabolic syndrome prevalence and were associated with most of its components in both sexes, except for the association between GGT and low high-density lipoprotein (HDL) cholesterol in men. Stratified analyses by alcohol drinking status revealed that within the same tertile category of serum ALT and GGT, subjects classified as alcohol abstainers showed higher adjusted odds ratios for metabolic syndrome prevalence than those classified as regular alcohol drinkers in both sexes. The interaction effects of serum GGT with alcohol drinking status on metabolic syndrome prevalence were significant in both sexes.

**Conclusions:**

These results suggest that elevated serum ALT and GGT, even within the reference range, are independently associated with increased metabolic syndrome prevalence, especially in alcohol abstainers, in Japanese men and women.

## Introduction

Metabolic syndrome, characterized as a cluster of metabolic disorders including central obesity, dyslipidemia, hypertension, and glucose intolerance, has been increasing in the developed countries [1], [2]. Subjects with metabolic syndrome are recognized to be at high risk of type 2 diabetes [3], [4] and cardiovascular diseases [5]. Because cardiovascular disease is one of the major causes of death, preventing cardiovascular damage is an important public health issue throughout the world. Early detection and early countermeasures against metabolic syndrome can help prevent the progression of cardiovascular damage.

Recent epidemiological and clinical studies have suggested that elevated serum levels of alanine aminotransferase (ALT) and gamma-glutamyl transferase (GGT), surrogate markers for liver injury, are associated with increased risk of metabolic syndrome, independent of their conventional risk factors [6]–[8]. However, little is known about the combined associations of elevated serum ALT and GGT with metabolic syndrome prevalence. Moreover, because regular alcohol drinkers tend to have higher circulating levels of hepatic enzymes, especially GGT, than abstainers, alcohol drinking status might have a modifying effect on the association between hepatic enzymes and the risk of metabolic syndrome. In this study, we evaluated the independent and combined associations of elevated serum levels of ALT and GGT with the prevalence of metabolic syndrome (and its components) in Japanese men and women. We further assessed whether alcohol drinking status had a modifying effect on these associations.

## Materials and Methods

### Ethics Statement

Participation in this study was essentially voluntary, and after the survey details were explained, written informed consent was obtained from each participant. As for the minors, written informed consent was obtained from both the person himself (herself) and his (her) guardian. The study protocol was reviewed and approved by the ethics committees of the Ministry of the Environment, Government of Japan, and the University of Tokushima Graduate School.

### Study subjects

This study was conducted using a dataset from the participants in the Survey on the Accumulation of Dioxins and Other Chemical Compounds in Humans, which has been conducted in Japan under the supervision of the Ministry of the Environment of Japan since 2002. The participant recruitment details have been previously reported [9], [10]. Briefly, we divided the whole of Japan into 5 regional blocks and selected a single prefecture as a survey area from each regional block every survey year. An open call for participants was performed through a public relations magazine, a poster, a local government office website, and broadcast in the area. A total of 2,266 participants (1,063 men and 1,203 women) aged 15–76 years were recruited from 2002–2010. We excluded the subjects who self-reported a history of stroke (n = 5), myocardial infarction (n = 11), hepatitis (n = 24), or cancer (n = 43). There were no subjects who self-reported a history of cirrhosis or hepatic cancer. We further excluded the subjects with missing hepatic enzyme data (n = 4). We finally analyzed 2,179 subjects (1,027 men and 1,152 women).

### Questionnaire

The participants were requested to complete a questionnaire on individual characteristics including smoking and alcohol drinking habits, dietary habits, and history of diseases and treatments. For drinking habits, subjects were asked how often they had consumed beer, sake, shochu (rough distilled spirits), and whisky according to 5 categories: almost every day, 3–4 times/week, 1–2 times/week, 1–2 times/month, and almost never. These 5 categories were converted to 6, 3.5, 1.5, 0.35, and 0.1 times/week, respectively. The frequency of alcohol drinking was summarized as the sum of the frequency of each item and then divided into 3 categories: daily, ≥6 times/week; often, 1.5 to <6 times/week; and rarely or never, <1.5 times/week. Regarding dietary habits, subjects were also asked how often they had consumed food items according to the same 5 categories as alcohol drinking and replaced with 6, 3.5, 1.5, 0.35, and 0.1 times/week, respectively. We did not specify the unit or portion of food. The food items were divided into five groups: meat-and-eggs (5 items: beef; pork; ham and sausage; bacon; and eggs); dairy products (4 items: milk; cheese; yogurt; and butter); fish-and-shellfish (7 items: coastal fishes such as house mackerel, mackerel, and sardine; other fishes such as tuna, salmon, and bonito; cuttlefish and octopus; crab; shrimp; tubular roll of grilled fish paste; and short-neck clam and corbicula); vegetables (2 items: green-yellow leafy vegetables; and other green-yellow vegetables); and fruit (1 item). The frequency of food group intake was summarized as the sum of the frequencies of food items included in each group. As for rice, subjects were asked how many bowls of rice they took on an average day, and never was expressed as 0.1 bowls/day.

### Measurements

Fasting venous blood was obtained from each participant, and biochemical factors ALT, GGT, high-density lipoprotein (HDL) cholesterol, triglycerides, and hemoglobin A1c (HbA1c) in the blood were measured with an automatic analyzer (model 7450; Hitachi, Tokyo, Japan). Blood pressure was measured in each subject sitting at rest. If a measurement deviated too far from normal values, we tried again after a short rest. A body mass index (BMI) was calculated as weight (in kilograms) divided by height (in meters) squared.

### Assessment of metabolic syndrome

We assessed the prevalence of metabolic syndrome using a modification of the National Cholesterol Education Program Adult Treatment Panel III definition (NCEP ATP-III 2005) [11]. It was reported that BMI was strongly correlated with waist circumference and the most accurate BMI cutoff point for abnormal waist circumference (≥90 cm in men and ≥80 cm in women used in the NCEP ATP-III definition) was 24.7 kg/m^2^ for men and 24.9 kg/m^2^ for women [12]. Therefore, we diagnosed metabolic syndrome if ≥3 of the following 5 criteria were satisfied: a BMI ≥25 kg/m^2^ instead of an abdominal waist circumference; serum triglycerides ≥150 mg/dL; serum HDL cholesterol <40 mg/dL in men or <50 mg/dL in women; a systolic blood pressure ≥130 mmHg and/or a diastolic blood pressure ≥85 mmHg, or a self-reported history of physician-diagnosed hypertension; and an HbA1c ≥5.2% (determined by the Japan Diabetes Society method) instead of fasting serum glucose, or a self-reported history of physician-diagnosed diabetes. An HbA1c of 5.2% determined by the Japan Diabetes Society method is equal to a value of 5.6% determined by the National Glycohemoglobin Standardization Program [13].

### Statistical analyses

Continuous variables are expressed as mean ± SD or median (25th percentile, 75th percentile). Categorical variables are expressed as proportion (percentage). Student’s *t*-test, Wilcoxon rank sum test, or Fisher’s exact test was used to compare the baseline characteristics between sexes.

The serum levels of ALT and GGT as well as the prevalence of metabolic syndrome exhibited considerable gender differences, and we found statistically significant interactions between gender and serum levels of ALT and GGT on metabolic syndrome prevalence (data not shown). Therefore, we analyzed the associations between ALT and GGT serum levels and the prevalence of metabolic syndrome and its components in logistic regressions in men and women separately, crude and adjusted for age (<40, 40 to <50, 50 to <60, and ≥60 years), regional blocks (Hokkaido/Tohoku, Kanto/Koshinetsu, Tokai/Hokuriku/Kinki, Chugoku/Shikoku, and Kyushu/Okinawa), smoking status (current, past, and never), alcohol drinking (daily, often, and rarely or never), and intakes of rice, meat-and-eggs, dairy products, fish-and-shellfish, vegetables, and fruit (continuous, log-transformed). Serum ALT and GGT levels were divided into tertiles in men and women so that the numbers of subjects in the 3 categories were almost equal, and the lowest category was used as the referent in the analyses. Dummy variables for categorical variables were created and, except for reference categories, were included in the model. Odds ratios and profile likelihood confidence intervals are presented. Tests of trend were assessed by assigning the ordinal categorical variables of 1, 2, and 3 for each tertile of the ALT and GGT serum levels in logistic models. We also conducted joint analyses by combining tertiles of ALT with those of GGT (9 categories in total) using the same logistic regression models in men and women separately. In these joint analyses, the combined category of the lowest ALT and the lowest GGT was used as the referent. We further evaluated associations of serum ALT or GGT with the metabolic syndrome prevalence using the logistic models stratified by alcohol drinking status (regular or non-regular drinker) to assess whether alcohol drinking had a modifying effect on the association between serum ALT or GGT and the metabolic syndrome prevalence. In these analyses, a combined category of the lowest ALT or GGT and non-regular drinking was used as the referent, and an alcohol intake frequency ≥1.5 times/week was defined as regular alcohol drinking. Interaction terms of the 2 exposure variables (serum ALT or GGT, continuous; alcohol drinking status, dichotomous) were created and added to the model to assess a statistical interaction.

All statistical analyses were performed with the SAS software package (version 8.2, SAS Institute Inc., Cary, NC). All *P* values are 2-tailed, and those <0.05 were considered statistically significant.

## Results

### Baseline characteristics

The baseline characteristics of the study subjects are shown in Table 1. Men showed significantly higher BMI, systolic and diastolic blood pressure, and serum triglycerides and significantly lower serum HDL cholesterol than women. Plasma HbA1c levels were not different between the sexes. Moreover, men showed significantly higher serum levels of ALT and GGT than women. The metabolic syndrome prevalence rates were 20.9% in men and 12.3% in women, indicating a significant sex disparity (*P* < 0.001).

**Table 1:** Characteristics of the study subjects.

	Men (No. = 1,027)		Women (No. = 1,152)		*P*
Age (years)^a^	43.1	(13.5)	44.8	(14.0)	0.003
BMI (kg/m^2^)^b^	23.4	(21.5, 25.8)	21.6	(19.9, 23.9)	<0.001
ALT (IU/L)^b^	23	(17, 35)	16	(12, 21)	<0.001
GGT (IU/L)^b^	33	(21, 57)	16	(12, 23)	<0.001
Systolic BP (mmHg)^a^	130.2	(18.7)	122.8	(20.0)	<0.001
Diastolic BP (mmHg)^a^	79.5	(12.9)	73.3	(11.8)	<0.001
Triglycerides (mg/dL)^b^	103	(73, 155)	72	(51.5, 104)	<0.001
HDL cholesterol (mg/dL)^a^	57.0	(14.8)	67.9	(15.3)	<0.001
HbA1c (JDS value) (%)^a^	5.05	(0.63)	5.02	(0.47)	0.144
Smoking habit^c^					<0.001
Current	388 (37.8)		71 (6.2)		
Past	250 (24.3)		66 (5.7)		
Never	387 (37.7)		1008 (87.5)		
Unknown	2 (0.2)		7 (0.6)		
Drinking habit^c^					<0.001
Daily	411 (40.0)		116 (10.1)		
Often	263 (25.6)		216 (18.8)		
Rarely or never	353 (34.4)		820 (71.2)		
Metabolic syndrome prevalence^c^					<0.001
Yes	215 (20.9)		142 (12.3)		
No	797 (77.6)		991 (86.0)		
Unknown	15 (1.5)		19 (1.6)		

a Mean ± SD, ^b^ Median (25%, 75%), ^c^ No. (%).

ALT, alanine aminotransferase; GGT, gamma-glutamyl transferase.

BP, blood pressure; HbA1c, hemoglobinA1c; JDS, Japan Diabetes Society.

### Associations of serum ALT and GGT with the metabolic syndrome prevalence

**Figure 1:**
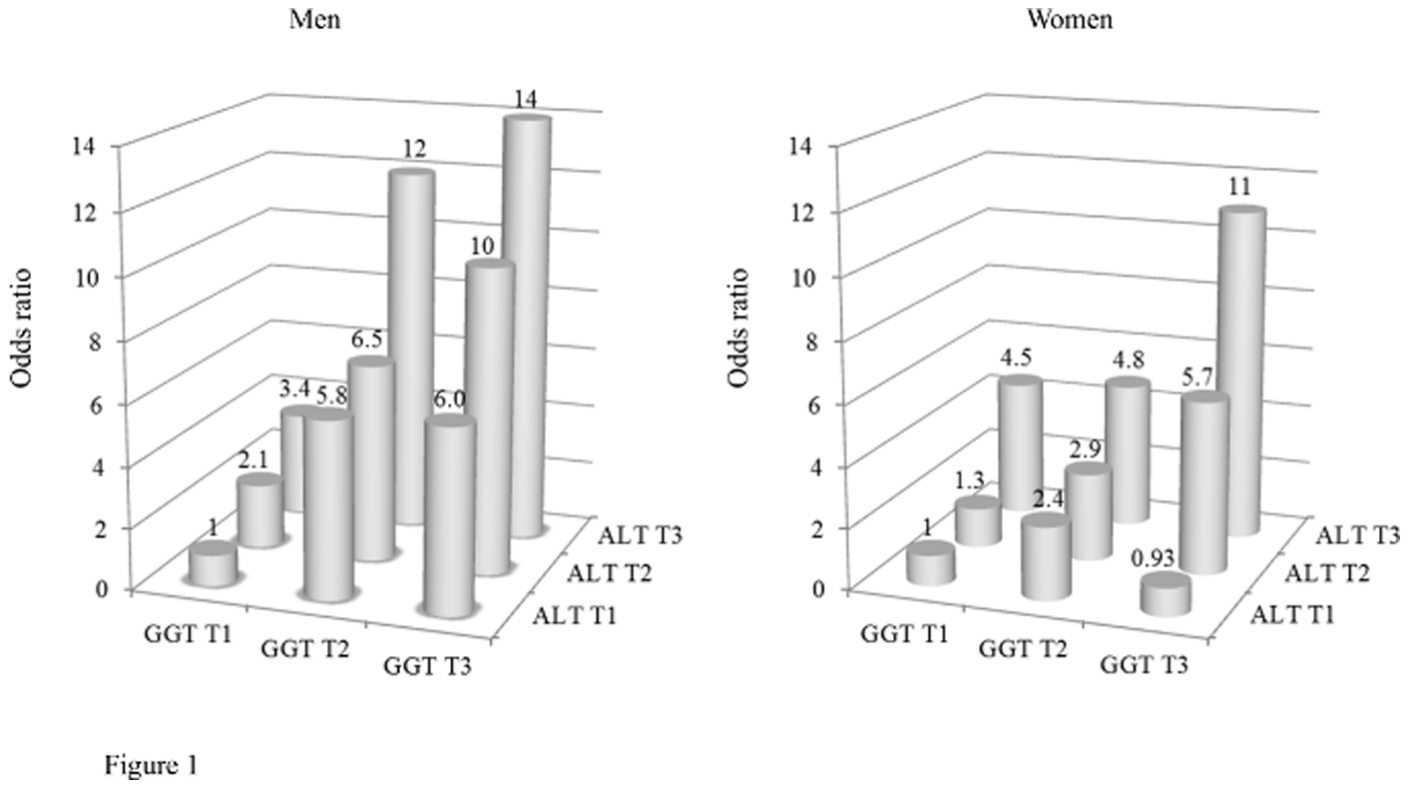
Combined associations of elevated serum ALT and GGT levels with metabolic syndrome prevalence in men and women. Estimated odds ratios for the metabolic syndrome prevalence adjusted for age, smoking habit, alcohol drinking, and intakes of rice, meat-and-eggs, dairy products, fish-and-shellfish, vegetables, and fruit are indicated. Abbreviations: ALT, alanine aminotransferase; GGT, gamma-glutamyl transferase; T1, lowest tertile; T2, middle tertile; T3, highest tertile.

Table 2 shows the associations between elevated ALT and GGT serum levels and the prevalence of metabolic syndrome and its components in men and women. The ALT and GGT serum levels showed proportional positive crude and adjusted associations with the metabolic syndrome prevalence in both sexes (tests of trend were all significant). In particular, the middle tertiles of serum ALT as well as that of GGT, even within the reference range, showed significantly higher adjusted odds ratios for the prevalence of metabolic syndrome in both sexes compared with the respective lowest tertile. As presented in Figure 1, an elevation in serum ALT was positively associated with the metabolic syndrome prevalence in a dose-dependent manner within each tertile of serum GGT in both sexes. Similarly, an elevation in serum GGT was also positively associated with the metabolic syndrome prevalence in a dose-dependent manner within each tertile of serum ALT, except for the lowest tertile of ALT in women. As shown in Table 2, the ALT and GGT serum levels were proportionally and positively associated with most of the metabolic syndrome components in both sexes, except for the association between GGT and low HDL cholesterol in men.

**Table 2:** Associations between elevated serum levels of ALT and GGT and prevalence of metabolic syndrome and its components in men and women.

	Men							Women					
	ALT (IU/L)				GGT (IU/L)			ALT (IU/L)			GGT (IU/L)		
	≤19	>19 to 30		>30	≤24	>24 to 47	>47	≤13	>13 to 19	>19	≤14	>14 to 20	>20
Metabolic syndrome													
Prevalence (%)	11.0	20.9		33.2	6.8	24.5	33.3	2.7	10.0	27.1	4.2	9.8	26.2
OR (95%CI)^a^	1	2.1 (1.4-3.3)		4.0 (2.7-6.1)	1	4.4 (2.8-7.3)	6.8 (4.3-11)	1	4.0 (2.1-8.3)	13 (7.3-27)	1	2.5 (1.4-4.5)	8.1 (4.9-14)
*P*_trend_	<0.001				<0.001			<0.001			<0.001		
OR (95%CI)^b^	1		2.3 (1.5-3.6)	4.9 (3.2-7.6)	1	5.3 (3.3-9.4)	8.6 (5.1-15)	1	2.2 (1.1-4.8)	6.1 (3.2-13)	1	2.0 (1.1-3.9)	5.0 (2.9-9.1)
*P*_trend_	<0.001				<0.001			<0.001			<0.001		
BMI ≥25 kg/m^2^													
Prevalence (%)	15.7		31.6	51.3	16.5	36.8	44.0	8.4	16.9	30.2	9.9	17.5	28.5
OR (95%CI)^b^	1		2.6 (1.8-3.8)	6.1 (4.2-9.0)	1	3.7 (2.5-5.4)	5.6 (3.7-8.4)	1	2.0 (1.2-3.2)	3.5 (2.2-5.6)	1	1.6 (1.1-2.5)	2.5 (1.7-3.9)
*P*_trend_	<0.001				<0.001			<0.001			<0.001		
Triglycerides ≥150 mg/dL													
Prevalence (%)	14.8		26.9	39.6	10.1	26.2	44.8	2.7	8.7	20.2	3.5	7.9	20.7
OR (95%CI)^b^	1		2.1 (1.4-3.1)	3.9 (2.7-5.8)	1	3.1 (2.0-4.9)	7.8 (5.0-12)	1	2.3 (1.1-5.2)	5.5 (2.7-12)	1	2.3 (1.2-4.7)	5.4 (3.0-11)
*P*_trend_	<0.001				<0.001			<0.001			<0.001		
HDL cholesterol <40 mg/dL in men and <50 mg/dL in women													
Prevalence (%)	5.9		6.7	12.1	7.3	11.6	5.4	6.3	10.4	13.8	7.0	8.7	15.2
OR (95%CI)^b^	1		1.2 (0.60-2.2)	2.4 (1.4-4.4)	1	2.0 (1.2-3.6)	1.0 (0.50-1.9)	1	1.5 (0.88-2.7)	1.9 (1.1-3.4)	1	1.2 (0.67-2.0)	2.2 (1.3-3.7)
*P*_trend_	0.003				0.829			0.036			0.004		
SBP ≥130 mmHg or DBP ≥85 mmHg or hypertension treatment													
Prevalence (%)	46.9		55.2	59.1	41.6	55.1	64.8	23.5	34.9	53.2	23.5	36.9	52.7
OR (95%CI)^b^	1		1.5 (1.1-2.0)	1.9 (1.3-2.6)	1	1.7 (1.2-2.3)	2.1 (1.5-3.0)	1	1.1 (0.79-1.6)	1.9 (1.3-2.7)	1	1.6 (1.2-2.3)	2.2 (1.5-3.1)
*P*_trend_	<0.001				<0.001			<0.001			<0.001		
HbA1c (JDS value) ≥5.2%* or diabetes treatment													
Prevalence (%)	24.7		32.3	32.1	20.2	30.3	38.7	15.7	28.1	45.1	20.4	27.4	41.3
OR (95%CI)^b^	1		1.5 (1.0-2.1)	1.7 (1.1-2.4)	1	1.8 (1.2-2.7)	2.4 (1.6-3.3)	1	1.4 (0.93-2.0)	2.5 (1.6-3.5)	1	1.2 (0.83-1.7)	1.8 (1.2-2.5)
*P*_trend_	0.008				<0.001			<0.001			0.002		

ALT, alanine aminotransferase; GGT, gamma-glutamyl transferase; OR, odds ratio; BP, blood pressure; HbA1c, hemoglobinA1c; JDS, Japan Diabetes Society.

^*^ An HbA1c of 5.2% determined by the JDS method is equal to a value of 5.6% determined by the National Glycohemoglobin Standardization Program.

a crude; ^b^ adjusted for age, regional blocks, smoking habit, alcohol drinking, and intakes of rice, meat-and-eggs, dairy products, fish-and-shellfish, vegetables, and fruit.

### Combined associations of serum ALT and GGT with alcohol drinking status on metabolic syndrome prevalence

As shown in Table 3, within the middle and highest tertiles of serum ALT and GGT in men and within the highest tertile in women, non-regular drinkers showed considerably higher adjusted odds ratios for metabolic syndrome prevalence than regular drinkers. The interaction effects between serum GGT and alcohol drinking status on metabolic syndrome prevalence were significant in both sexes (*P* values for interaction were 0.022 in men and <0.001 in women, respectively). Meanwhile, the interaction effects between serum ALT and alcohol drinking status were not significant in both sexes.

**Table 3:** Combined associations of elevated serum levels of ALT or GGT and alcohol drinking status with prevalent metabolic syndrome.

	Regular alcohol drinking					Regular alcohol drinking			
	No		Yes			No		Yes	
	a OR	(95%CI)	a OR	(95%CI)		a OR	(95%CI)	a OR	(95%CI)
Men					Women				
ALT (IU/L)					ALT (IU/L)				
≤19	1		0.73	(0.37-1.6)	≤13	1		0.94	(0.20-3.5)
>19 to 30	2.2	(1.0-4.8)	1.7	(0.91-3.5)	>13 to 19	2.3	(1.0-5.7)	1.9	(0.72-5.4)
>30	5.7	(2.8-12)	3.3	(1.7-6.7)	>19	6.5	(3.1-16)	4.8	(2.0-12)
b *P*_interaction_	0.565				b *P*_interaction_	0.090			
GGT (IU/L)					GGT (IU/L)				
≤24	1		0.77	(0.32-1.8)	≤14	1		0.71	(0.16-2.3)
>24 to 47	8.9	(4.4-19)	2.5	(1.3-5.1)	>14 to 20	1.9	(0.91-3.8)	1.9	(0.74-4.7)
>47	8.2	(3.8-19)	4.7	(2.5-9.5)	>20	5.2	(2.8-10)	2.9	(1.4-6.2)
c *P*_interaction_	0.022				c *P*_interaction_	<0.001			

ALT, alanine aminotransferase; GGT, gamma-glutamyl transferase.

a adjusted for age, regional blocks, smoking status, and intakes of rice, meat-and-eggs, dairy products, fish-and-shellfish, vegetables, and fruit.

b P for interaction between ALT (continuous) and alcohol drinking status(dichotomous).

c P for interaction between GGT (continuous) and alcohol drinking status(dichotomous).

When we excluded the subjects who self-reported a history of physician-diagnosed diabetes from the analyses (n = 85), none of the results were substantially altered.

## Discussion

In this study, we investigated independent proportional associations of serum ALT and GGT elevation, even within the reference range, with the prevalence of metabolic syndrome and most of its components in both sexes. Within similar serum levels of ALT and GGT, abstainers may be at increased prevalence of metabolic syndrome compared with regular alcohol drinkers.

In recent years, associations between elevated serum levels of liver enzymes and increased cardiovascular risk have drawn attention. Elevations in serum ALT and GGT have been shown to predict cardiovascular events in prospective studies independently from conventional cardiovascular risk factors [14]–[16]. Metabolic syndrome, recognized to be a high risk for cardiovascular diseases, has been increasing in Asian countries, including Japan, with the westernization of lifestyle. The co-occurrence of several components of the metabolic syndrome may have a considerably greater impact on cardiovascular events than any one alone [17]. In the present study, we revealed that elevated serum ALT and GGT levels, even within the reference range, are proportionally associated with an increased metabolic syndrome prevalence in men and women. Several studies have demonstrated an association between prevalent nonalcoholic fatty liver disease (NAFLD) and elevated serum ALT [18], [19]. The clinically most common cause of elevated serum ALT is NAFLD, rather than alcohol consumption or viral hepatitis, in the developed countries [20], [21]. ALT is demonstrated to be most closely related to liver fat accumulation among the hepatic enzymes [22]. NAFLD is highly related to obesity, hypertension, dyslipidemia, and insulin resistance, which are constituent features of the metabolic syndrome [23], [24]. Therefore, NAFLD is considered a hepatic manifestation of metabolic syndrome. Elevated serum GGT, which contributes to extracellular glutathione catabolism [25], has also been widely used as a marker for liver dysfunction. GGT is widely distributed in the human body, and in recent years, epidemiological studies have demonstrated various physiological effects of elevated serum GGT, irrespective of alcohol consumption. Elevated serum GGT has been demonstrated to be a predictor of metabolic syndrome incidence [15], and this corroborates our cross-sectional finding. The mechanisms underlying the relationship between serum GGT elevation and metabolic syndrome prevalence are not fully verified, although central obesity and liver fat accumulation are possible pathways [26], [27]. In addition to these conditions, serum GGT is elevated in a number of diseases characterized by oxidative stress as part of their pathogenesis, and elevated GGT activity may be a response to oxidative stress. Serum GGT is also elevated in several diseases characterized by chronic inflammation, such as cardiovascular diseases, and GGT is suggested to be an inflammatory marker [28]. Although associations between elevated serum hepatic enzymes and metabolic syndrome prevalence are thought to be mediated by several pathways, ALT may be mainly related to liver fat accumulation and GGT may be broadly related to liver fat accumulation, oxidative stress, and inflammation.

The upper limits of the reference range of serum hepatic enzymes differ somewhat by laboratories according to the commercial kit used and the reference population. The upper limit of the reference range of ALT is generally approximately 40 U/L [29], including for the Japanese. However, ALT levels in some subjects, especially women, with clinical or subclinical liver disease including NAFLD, cannot be detected with this upper limit. Updated upper limits of healthy ranges of ALT for men and women were suggested to be 30 and 19 U/L, respectively [30]. Coincidentally, the cutoff levels between the middle and the highest tertiles of serum ALT in men and women in the current study entirely agreed with these suggested upper limits. In this study, subjects whose serum levels of ALT were higher than these suggested upper limits (i.e., the subjects in the highest tertiles) had significantly higher odds ratios of 4.9 and 6.1 for metabolic syndrome prevalence compared with those in the lowest tertiles in men and women, respectively; therefore, these suggested upper limits may be acceptable. Similarly, as for serum GGT levels, subjects whose serum levels of GGT were higher than 47 U/L and 20 U/L (i.e., the subjects in the highest tertiles), being lower than the recognized upper limits of the reference range of GGT, had significantly higher odds ratios of 8.6 and 5.0 for metabolic syndrome prevalence compared with those in the lowest tertiles in men and women, respectively. Therefore, the historically established upper limits of the reference range of GGT in both sexes are also thought to be rather high.

We further investigated that elevated serum ALT and GGT levels were independently associated with the metabolic syndrome prevalence in a dose-dependent manner in both sexes (Figure 1). We found that subjects with elevated serum ALT and GGT levels, even within the reference range, were associated with increased prevalence of most of the components of metabolic syndrome in both sexes. If serum elevations of both ALT and GGT would be associated with similar limited components of the metabolic syndrome, or if their associations with metabolic syndrome prevalence would be mediated by similar mechanisms, we could not investigate their independent associations. However, serum elevations of both ALT and GGT were broadly associated with most of the components of metabolic syndrome, probably through different main mechanisms, with ALT mainly related to liver fat accumulation and GGT mainly related to oxidative stress and inflammation. Therefore, we could investigate their independent associations.

Generally, serum hepatic enzymes, especially GGT, increase as alcohol consumption increases [31]. We found that subjects who were not regular drinkers showed an increased metabolic syndrome prevalence compared with regular drinkers within similar levels of serum ALT and GGT beyond approximately 20 IU/L, even within the reference range, in both sexes. The physiology underlying this finding cannot be entirely explained. One probable explanation is that the associations between elevated serum liver enzymes and metabolic syndrome prevalence are independent of alcohol drinking but that regular drinkers exhibit higher levels of serum GGT and ALT than abstainers among the subjects having a similar risk of metabolic syndrome. In turn, abstainers have an increased risk of metabolic syndrome compared with regular drinkers among the subjects showing similar levels of serum GGT and ALT. Another explanation may be that alcohol consumption is related to a reduced risk of metabolic syndrome. Alkerwi et al. have suggested by a meta-analysis that responsible alcohol intake (<20 g/day among women and <40 g/day among men) appears to be associated with a reduced metabolic syndrome prevalence [32]. Stoutenberg et al. further reported that alcohol consumption was inversely associated with metabolic syndrome incidence [33]. In contrast, several reports have demonstrated a link between alcohol consumption and increased risk of metabolic syndrome [34], [35]. A detailed prospective study including information on the amount of alcohol consumption and types of alcoholic beverages is needed to clarify the influence of drinking alcohol on the associations between serum GGT or ALT and the risk of metabolic syndrome.

The strength of the present study is that our subjects represent the general population of Japan. However, this study has several limitations. First, owing to the nature of the cross-sectional study design, causal relationships between elevated serum ALT or GGT levels and metabolic syndrome prevalence should be interpreted with caution. Second, we diagnosed metabolic syndrome using a criterion of BMI ≥25 kg/m^2^ instead of waist circumference, but BMI may not rigorously reflect central obesity. Similarly, we used HbA1c as a criterion instead of fasting serum glucose because we did not have data on fasting serum glucose in all subjects. Third, information on alcohol drinking and other lifestyle factors was based on self-reporting, and nondifferential misclassification may be inevitable, and this misclassification might attenuate the observed associations. Fourth, we did not measure physical activities which could modify the relationship between serum hepatic enzyme levels and/or alcohol consumption and metabolic syndrome. Finally, because all of our subjects were Japanese, our results may not be applicable to other ethnic populations.

## Conclusions

These representative data of the Japanese general population suggest that elevated serum levels of ALT and GGT, even within the reference range, are independently associated with an increased metabolic syndrome prevalence in both sexes. Within similar serum levels of ALT and GGT, abstainers may be at increased prevalence of metabolic syndrome compared with non-regular alcohol drinkers in both sexes. Further epidemiologic studies using longitudinal designs should be conducted to define the causality between elevated serum ALT or GGT levels and metabolic syndrome.
